# Diagnostic Effect of Attenuation Correction in Myocardial Perfusion Imaging in Different Coronary Arteries: A Systematic Review and Meta-Analysis

**DOI:** 10.3389/fcvm.2021.756060

**Published:** 2021-10-12

**Authors:** Jei-Yie Huang, Chun-Kai Huang, Ruoh-Fang Yen, Kuo-Liong Chien, Yen-Wen Wu

**Affiliations:** ^1^Department of Nuclear Medicine, National Taiwan University Hospital, National Taiwan University College of Medicine, Taipei, Taiwan; ^2^Institute of Epidemiology and Preventive Medicine, College of Public Health, National Taiwan University, Taipei, Taiwan; ^3^Department of Internal Medicine, National Taiwan University Hospital, National Taiwan University College of Medicine, Taipei, Taiwan; ^4^Department of Nuclear Medicine, Far Eastern Memorial Hospital, New Taipei City, Taiwan; ^5^Division of Cardiology, Cardiovascular Medical Center, Far Eastern Memorial Hospital, New Taipei City, Taiwan; ^6^National Yang Ming Chao Tung University School of Medicine, Taipei, Taiwan

**Keywords:** attenuation correction, myocardial perfusion imaging, single photon emission computed tomography, coronary artery, meta-analysis

## Abstract

**Background:** The aim of this study was to determine whether, and if so how, attenuation correction (AC) improves the diagnostic performance of myocardial perfusion imaging (MPI) in different coronary artery-supplied territories, using coronary angiography as the reference standard.

**Methods:** PubMed and EMBASE were searched until December 2020 for studies evaluating AC MPI for the diagnosis of coronary artery disease (CAD) with vessel-based data. Methodological quality was assessed using the Quality Assessment of Diagnostic Accuracy Studies tool. For each study, the sensitivity, specificity, diagnostic odds ratios and areas under summary receiver operating characteristic curves (AUC) with 95% confidence intervals were calculated to determine the diagnostic accuracy of AC compared to non-AC MPI. A bivariate mixed-effects model was used to pool the data. Subgroup analyses considering the type of radiotracer and type of AC were performed.

**Results:** A total of 264 articles were screened, of which 22 studies (2,608 patients) were enrolled. Significant improvements in specificity [0.78 vs. 0.58 in overall CAD, 0.87 vs. 0.61 in right coronary artery (RCA)] and diagnostic odds ratios (16 vs. 8 in overall CAD, 18 vs. 7 in RCA) after AC were shown in overall CAD at a patient level and RCA stenosis. Improvements in AUC were also noted. MPI had a similar diagnostic performance for detecting left anterior descending and left circumflex coronary artery stenosis with or without AC. There were trends of decreased sensitivity after AC, but none were significant. Diagnostic odds ratio showed significant improvement after AC only in the technetium-99m subgroup.

**Conclusion:** The results of this study suggest that AC should be applied to MPI to improve the diagnosis of CAD regardless of which type of radiotracer, and that AC MPI can improve the specificity of detecting RCA stenosis.

## Introduction

Coronary artery disease (CAD) is a major cause of mortality and morbidity worldwide. Mortality and morbidity due to CAD are associated with the number of territories at risk, and are highest for left main and proximal left anterior descending (LAD) coronary artery lesions, lowest for right coronary artery (RCA) disease, and intermediate for non-proximal LAD and left circumflex artery (LCX) disease (1–4). Choosing the correct target vessel for intervention is crucial when performing coronary angiography (CAG) and interventional procedures, especially in multi-vessel disease (5–7).

Myocardial perfusion imaging (MPI) with single photon emission computed tomography (SPECT) is a well-established diagnostic tool for CAD, and it has been shown to have value in treatment decision-making before CAG (8, 9). However, attenuation artifacts may cause misinterpretation and decrease the diagnostic accuracy (10). Attenuation artifacts in MPI are caused by the different densities of various thoracic tissues, resulting in different attenuation patterns in different coronary territories (10). Although attenuation correction (AC) was developed and suggested to overcome this artifact, was not routinely done in clinical practice (11). Also, the performance of AC is different in each part of the myocardium, and sex differences also exist (12). The magnitude of the attenuation effect are less intense at higher energy radiotracers (10). Whether higher energy radiotracer like technetium-99m [^99m^Tc] needed AC is a question.

Previous studies have evaluated the effect of AC in the patient-based diagnostic performance for CAD and assessed its effect in individual coronary arteries with stenosis. For example, some studies have reported that AC can increase the specificity of detecting RCA stenosis (13–15), however the results have been inconsistent with regards to LAD and LCX disease. One study reported no difference in specificity (13), another showed decreased specificity (15), and another showed increased specificity (14). Furthermore, another study showed increased diagnostic sensitivity in three vessel territories, increased specificity in the LAD and LCX, but no change in the RCA (16). Significant discordances were noted between these studies. Moreover, most MPI studies have been heterogeneous with rather small study populations.

Therefore, the aim of this study was to evaluate the diagnostic performance of MPI at a vessel-based level using a meta-analysis method, and compare AC and non–attenuation corrected (NAC) MPI, using CAG as the reference standard. In addition, subgroup analyses (considering the type of radiotracer, ^99m^Tc vs. thallium-201 [^201^Tl], radionuclide source AC [RAC] vs. computed tomography AC [CTAC] and definition of CAD, 50% vs. 70% stenosis) were performed.

## Materials and Methods

### Search Strategy

PubMed and EMBASE were searched for English-language and human studies from inception to 1st December 2020 for studies evaluating AC MPI for the diagnosis of CAD with vessel-based data provided. The search keywords included myocardial perfusion, SPECT, and AC. Conference articles were excluded as most did not provide precise data [i.e., true positives (TP), false positives (FP), false negatives (FN), and true negatives (TN)]. Medical Subject Headings terms were used to maximize the sensitivity of the search.

### Data Extraction

The search and selection strategies were carried out in accordance with the Preferred Reporting Items for Systematic Reviews and Meta-Analyses (PRISMA) guidelines (17). The detailed review protocol was registered on PROSPERO (registration number: CRD42019124218) (18). Available from: https://www.crd.york.ac.uk/prospero/display_record.php?ID=CRD42019124218. Article titles and abstracts were reviewed for eligibility. The inclusion criteria were studies in which: (1) AC MPI was assessed as a diagnostic tool to evaluate patients for the presence of at least one coronary artery stenosis; (2) coronary artery stenosis was defined as ≥50% diameter stenosis on CAG; (3) CAG was used as the reference test; and (4) the absolute numbers of TP, FP, TN, and FN were available or these data were derivable from the results presented. The exclusion criteria were: (1) studies with duplicate subject enrollment; (2) technology, image quality, review, or continuing education studies; (3) studies with no diagnostic performance or vessel-based data provided; (4) studies that used a reference test other than CAG; (5) and studies in which TP, FP, TN, and FN data could not be extracted. We checked all references in the included articles manually, and articles which met the inclusion criteria were also enrolled.

### Data Extraction

Two researchers (JYH and CKH) independently performed data extraction. The extracted information included author, journal, year of publication, and country; details of the study design; patient demographic features (such as numbers of patients, mean age, percentage of males, and indication for MPI); imaging technique (such as type of AC, type of perfusion radiotracer, type of stress); imaging protocol (scatter correction, gated); brand of imaging device and interpretation method; definition of CAD; and numbers of TP, FP, TN, and FN at a patient level and at each coronary artery level. If a study reported more than one pair of sensitivities and specificities at different cutoff points, different imaging techniques, different CAD definitions, or different experienced interpreters, the pair reported in the abstract (19) and the pair with the highest sensitivity (16, 20) were extracted. Disagreements between the two researchers were resolved by consensus.

### Quality Assessment

Methodological quality was assessed using the Quality Assessment of Diagnostic Accuracy Studies (QUADAS; scale, 0–14) tool (21). We chose QUADAS due to its clearer definitions and answers to assessment questions. In brief, the QUADAS tool is comprised of 14 items: covered patient spectrum, reference standard, disease progression bias, verification bias, review bias, clinical review bias, incorporation bias, test execution, study withdrawals, and indeterminate results. A score of 7–14 is considered to be high quality, and a score below 7 low quality.

### Statistical Analysis

All data from each eligible study were extracted. Continuous variables were expressed as mean values, and categorical variables were expressed as percentages. On the basis of extracted 2 by 2 contingency tables, pooled estimates for diagnostic performance, including sensitivity, specificity, diagnostic odds ratio (DOR), summary receiver-operating characteristic (ROC) curve, and area under the curve (AUC) with 95% confidence intervals (CIs) were calculated. Between-study statistical heterogeneity was assessed using *I*^2^ and the Cochrane Q test on the basis of mixed-effects analysis (22). Publication bias was examined using the effective sample size funnel plot and associated regression test of asymmetry described by Deeks et al., with a *P*-value of <0.10 for the slope coefficient indicating significant asymmetry (23). Diagnostic performance estimates across studies and comparisons between different index tests were analyzed using a bivariate mixed-effects regression model at the patient level and at each coronary artery level (24, 25).

The assumption of the bivariate model is that the sensitivities and specificities (after logit transformation) from each individual study within the meta-analysis are approximately normally distributed around a mean value, with a certain amount of variability around this mean, resulting in bivariate normal distribution. We analyzed the bivariate model using linear mixed-model techniques. The parameters of the bivariate model were estimated in a single model to incorporate correlations between sensitivities and specificities. The summary ROC curves were also created using this model (24).

Additional subgroup analyses were performed, classified by the type of AC (CTAC or RAC), type of radiotracer (^201^Tl or ^99m^Tc) and definition of CAD (50% or 70% stenosis). All statistical analyses were performed using STATA (version 14; StataCorp LP, College Station, TX) and SAS (version 9.4; SAS Institute Inc., Cary, NC).

## Results

### Study Characteristics

**Figure 1:**
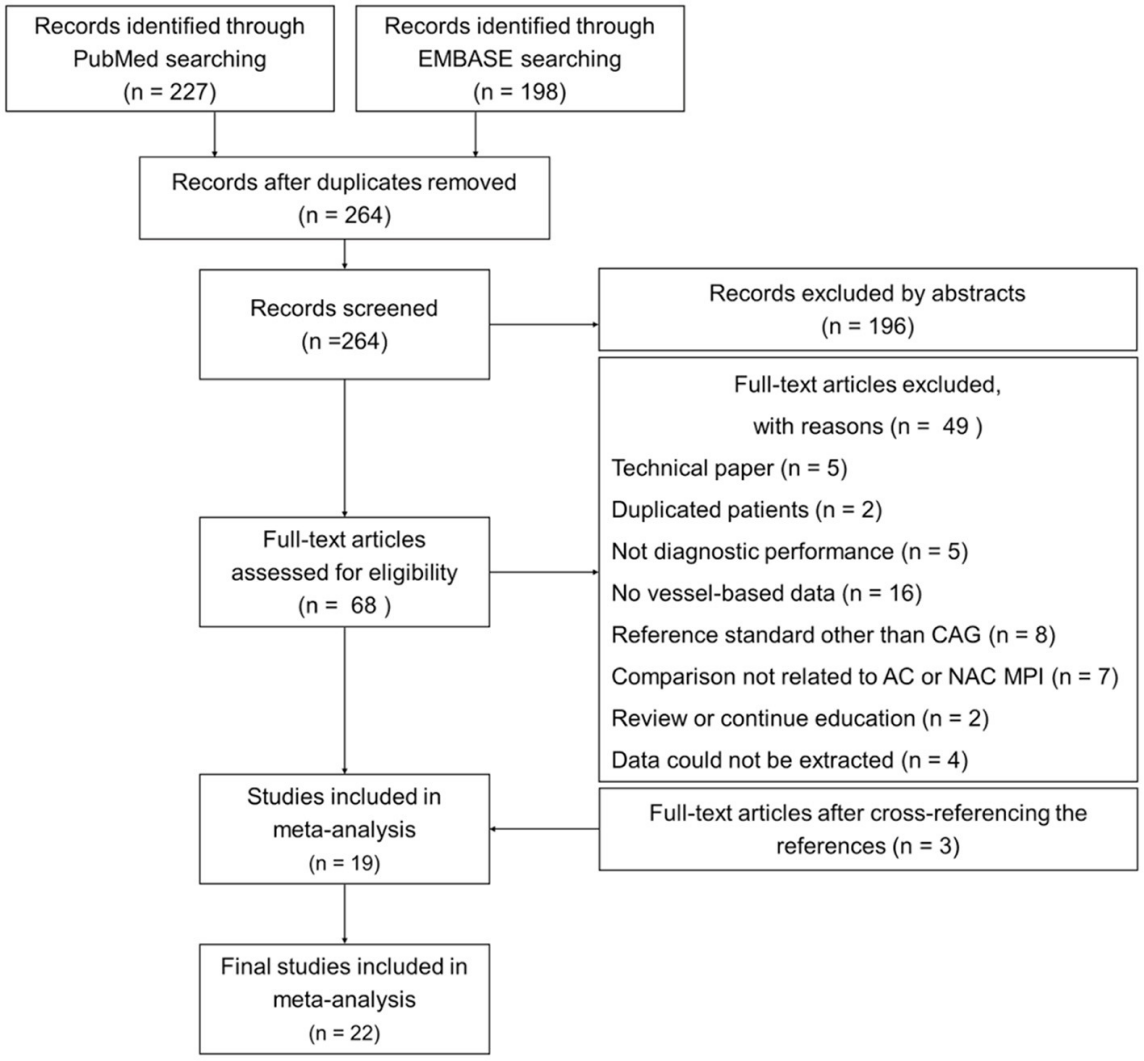
Flowchart of study selection. A total of 22 studies (2,608 patients) were included in the final analysis. AC, attenuation correction; CAG, coronary angiography; MPI, myocardial perfusion imaging; NAC, non-AC.

Two hundred and twenty-seven and 198 articles limited to humans and the English language and excluding conference articles were retrieved through the searches of PubMed and EMBASE, respectively (Figure 1). After excluding duplicate articles, 264 articles remained. On the basis of title and abstract, an additional 196 articles were excluded. After full text review, 49 additional articles were excluded, and the reasons are shown in Figure 1. Three studies were included after manually checking the reference lists (19, 26, 27). The flowchart of study selection is shown in Figure 1.

The final analysis included 22 studies (2,608 patients), of which 18 studies (2,428 patients) reported data on CAD at a patient-level, 21 studies (2,504 patients) reported data on LAD ischemia, 20 studies (2,448 patients) reported data on LCX ischemia, and 22 studies (2,606 patients) reported data on RCA ischemia. Five studies used ^201^Tl (13, 14, 28–30), 15 studies used ^99m^Tc, and two studies used dual radiotracers (19, 27). Twelve studies used CTAC, seven studies used RAC (19, 27, 29, 31–34), two studies used AC with segmentation of scatter and photo-peak window data (SSPAC) (35, 36), and one study used computer-generated Chang AC (30). The detailed characteristics of each study are shown in Table 1 (13–16, 19, 20, 26–41).

**Table 1:** Characteristics of the selected studies.

**References**	**Country**	***n***	**Age (SD)**	**% male**	**Type of AC**	**Tracer**	**Test interval**	**CAD definition**	**Pt AC Se/Sp**	**Pt NAC Se/sp**	**LAD AC Se/Sp**	**LAD NAC Se/sp**	**LCX AC Se/Sp**	**LCX NAC Se/sp**	**RCA AC Se/Sp**	**RCA NAC Se/sp**
Yamauchi et al. (36)	Japan	179	72 (8)	66	SSPAC	^99^mTc	3 mo	>50%	0.85/0.64	0.75/0.68	0.79/0.83	0.47/0.88	0.77/0.95	0.60/0.93	0.72/0.88	0.47/0.83
Huang et al. (14)	Taiwan	108	64 (11)	67	CTAC	^201^TI	3 mo	LM>50% >70%	0.79/0.78	0.82/0.57	0.93/0.56	0.93/0.39	0.70/0.76	0.73/0.59	0.73/0.70	0.84/0.48
Xin et al. (37)	China	181	62 (9)	68	CTAC	^99^mTc	3–6 mo	>50%	0.53/0.87	0.76/0.55	0.39/0.90	0.43/0.86	0.26/0.99	0.39/0.97	0.26/0.93	0.60/0.65
Konishi et al. (28)	Japan	34	70 (10)	97	CTAC	^201^TI	NA	Mix	NA/NA	NA/NA	0.90/0.91	0.73/0.83	0.90/0.83	0.90/0.88	0.80/0.86	0.80/0.84
Plachcinska et al. (38)	Poland	107	62 (8)	61	CTAC	^99^mTc	3 mo	LM>50% >70%	0.79/0.86	0.83/0.63	0.71/0.92	0.54/0.87	0.17/0.96	0.50/0.96	0.65/0.88	0.78/0.73
Caobelli et al. (39)	Germany	44	65 (11)	71	CTAC	^99^mTc	6 mo	≧70%	0.80/1.00	0.87/0.40	0.73/0.64	0.70/0.36	0.70/0.71	0.90/0.33	0.83/0.81	0.96/0.19
Yamauchi et al. (36)	Japan	150	70 (7)	68	SSPAC	^99^mTc	3 mo	>50%	0.91/0.90	0.77/0.76	0.93/0.85	0.71/0.91	0.81/0.98	0.70/0.98	0.92/0.92	0.92/0.67
Sharma et al. (20)	India	171	55 (10)	82	CTAC	^99^mTc	3 mo	>50%	0.57/0.89	0.65/0.83	0.51/0.91	0.49/0.90	0.27/0.93	0.32/0.89	0.26/0.95	0.43/0.89
Huang et al. (15)	China	99	61 (12)	65	CTAC	^99^mTc	60 d	>70%	0.92/0.79	0.95/0.63	0.94/0.82	0.87/0.94	0.91/0.91	0.86/0.94	0.73/0.99	0.91/0.78
Genovesi et al. (40)	Italy	104	64 (10)	79	CTAC	^99^mTc	1 mo	LM>50% >70%	0.75/0.81	0.82/0.93	NA/NA	NA/NA	NA/NA	NA/NA	0.72/0.99	0.80/0.71
Xu et al. (31)	U.S.	650	64 (12)	NA	^153^Gd	^99^mTc	60 d	>70%	0.84/0.75	0.83/0.65	0.75/0.81	0.73/0.78	0.57/0.77	0.56/0.75	0.65/0.81	0.70/0.78
Bajen et al. (41)	Spain	60	63 (NA)	75	CTAC	^99^mTc	3 mo	>50%	NA/0.86	NA/0.75	0.83/0.74	0.79/ 0.94	0.56/1.00	0.56/0.93	0.85/0.82	0.96/0.38
Utsunomiya et al. (13)	Japan	30	68 (NA)	60	CTAC	^201^TI	2 w	>50%	NA/NA	NA/NA	0.80/0.90	0.70/0.95	0.67/0.96	0.50/0.96	0.80/0.92	0.80/0.68
Masood et al. (16)	US	118	61 (12)	67	CTAC	^99^mTc	3 mo^*^	>50%	0.94/0.59	0.93/0.56	0.93/0.37	0.92/0.35	0.96/0.32	0.96/0.32	0.98/0.43	0.97/0.41
Grossman et al. (32)	U.S.	74	NA	NA	^153^Gd	^99^mTc	2 mo	≧70%	0.90/0.57	0.97/0.29	0.60/0.76	0.72/0.49	0.79/0.71	0.74/0.73	0.68/0.90	0.82/0.65
Banzo et al. (33)	Spain	99	59 (NA)	72	^153^Gd	^99^mTc	3 mo	>70%	0.76/0.71	0.92/0.46	0.70/0.74	0.75/0.82	0.64/0.94	0.72/0.92	0.79/0.85	0.96/0.44
Vidal et al. (29)	France	56	59 (10)	82	^99m^Tc	^201^TI	3 mo	>70%	NA/NA	NA/NA	0.43/0.82	0.68/0.77	NA/NA	NA/NA	0.72/0.89	0.87/0.34
Hendel et al. (34)	US	112	61 (12)	56	100 keV	^99^mTc	3 mo	>50%	0.78/0.44	0.76/0.50	0.57/0.70	0.63/0.63	0.36/0.87	0.30/0.85	0.53/0.73	0.78/0.35
Birkenfeld et al. (30)	UK	22	NA	86	Chang AC	^201^TI	3 mo	≧70%	0.94/0.50	0.94/0.17	0.73/0.71	0.73/0.71	0.33/1.00	0.33/1.00	0.75/0.80	0.75/0.70
Raza et al. (26)	Pakistan	38	56 (13)	87	CTAC	^99^mTc	NA	≧70%	0.66/0.78	1.00/0.11	0.57/0.94	0.62/0.88	0.71/0.58	0.93/0.46	0.56/0.79	0.89/0.10
Links et al. (27)	U.S.	112	(-)	68	^153^Gd, ^99m^Tc	Dual tracer^†^	Trial period	>50%	0.88/0.92	0.84/0.69	0.77/0.93	0.64/0.71	0.50/0.97	0.32/0.94	0.74/0.95	0.71/0.81
Ficaro et al. (19)	U.S.	60	63 (12)	63	^241^Am	Dual tracer^‡^	90 d	>50%	0.88/0.82	0.84/0.46	0.81/0.77	0.72/0.73	0.76/0.71	0.63/0.52	0.82/0.76	0.54/0.65

*AC, attenuation correction; CAD, coronary artery disease; CTAC, computed tomography AC; d, days; LAD, left anterior descending artery; LCX, left circumflex artery; LM, left main coronary artery, Mix, coronary angiography, computed tomography angiography and clinical data; mo, months; NA, not available; NAC, non-AC; RCA, right coronary artery; SD, standard deviation; Se, Sensitivity, Sp, Specificity; SSPAC, attenuation correction using segmentation of scatter and photo-peak window data; w, weeks*;

* *6 months before or 3 months after MPI*;

† *combination of ^99m^Tc, ^201^Tl, and ^99m^Tc/^201^Tl stress/rest*;

‡ *^99m^Tc/^201^Tl stress/rest*.

The methodological quality of the 22 studies was assessed using the QUADAS tool. All of the studies were scored above 7, indicating good quality (21). Most studies were found to have problems with unclear masking during interpretation of the reference test, masked reading of the index test, lack of reporting for un-interpretable results, and no explanation for withdrawals in QUADAS assessment, which may have resulted in bias.

The differences between the studies could potentially have affected the pooled diagnostic performance of MPI. The *I*^2^ index showed substantial heterogeneity with regards to sensitivity and specificity for all index tests. The highest was 95.92% for measuring the specificity of detecting LCX stenosis with CTAC, and the lowest was 39.22% for measuring the specificity of detecting LAD stenosis with RAC. To assess publication bias, we chose the performance in detecting RCA stenosis with all AC data. This was because attenuation and AC are known to affect the RCA territory the most, and also because all of the included studies reported data on detecting RCA stenosis. Funnel plots and regression tests showed a statistically non-significant *P*-value (*P* = 0.14) for the slope coefficient, indicating symmetry in the data and a low likelihood of publication bias (23).

### Diagnostic Performance

The pooled estimates representing diagnostic performance are shown in Table 2. There were no significant differences in pooled sensitivities across the studies, ranging from 0.64 to 0.82 for diagnosing CAD at a patient level or detecting LAD, LCX, or RCA stenosis. The pooled specificities across the studies were 0.78 (95% CI, 0.71–0.83), 0.82 (0.75–0.86), 0.74 (0.62–0.83), and 0.58 (0.48–0.67) for all AC, CTAC, RAC, and all NAC, respectively, when diagnosing CAD at a patient level; 0.81 (0.75–0.86), 0.82 (0.72–0.89), 0.80 (0.76–0.84), and 0.79 (0.70–0.85) for all AC, CTAC, RAC, and all NAC when detecting LAD stenosis; 0.91 (0.83–0.95), 0.89 (0.76–0.96), 0.92 (0.83–0.96), and 0.86 (0.76–0.92) for all AC, CTAC, RAC, and all NAC when detecting LCX stenosis; and 0.87 (0.82–0.91), 0.88 (0.79–0.93), 0.87 (0.82–0.90), and 0.61 (0.51–0.71) for all AC, CTAC, RAC, and all NAC when detecting RCA stenosis. The pooled DORs, which are regarded as being minimally affected by verification bias, were 16 (11–24), 15 (10–22), 16 (8–33), and 8 (6–12) for all AC, CTAC, RAC, and all NAC, respectively, when diagnosing CAD at a patient level; 13 (9–20)s, 15 (9–26), 11 (6–21), and 9 (6–13) for all AC, CTAC, RAC, and all NAC when detecting LAD stenosis; 18 (10–32), 15 (7–33), 20 (8–52), and 11 (6–20) for all AC, CTAC, RAC, and all NAC when detecting LCX stenosis; and 18 (11–29), 18 (9–36), 19 (10–37), and 7 (5–10) for all AC, CTAC, RAC, and all NAC when detecting RCA stenosis.

**Table 2:** Diagnostic performance of MPI, pooled sensitivity, specificity, diagnostic OR and area under the receiver operating characteristic curve of all AC, CTAC, RAC, and NAC in diagnosing CAD at a patient level and detecting LAD, LCX, and RCA stenosis.

		**Sensitivity**	***P*** **-value**	**Specificity**	***P*** **-value**	**DOR**	***P*** **-value**	**AUC**
PT	All AC	0.82 (0.76–0.87)	0.29	0.78 (0.71–0.83)	<0.001	16(11–24)	0.015	0.87 (0.84–0.89)
	CTAC	0.77 (0.66–0.85)	0.18	0.82 (0.75–0.86)	<0.001	15(10–22)	0.08	0.86 (0.83–0.89)
	RAC	0.85 (0.82–0.88)	0.38	0.74 (0.62–0.83)	0.12	16(8–33)	0.08	0.87 (0.84–0.90)
	All NAC	0.86 (0.81–0.90)	NA	0.58 (0.48–0.67)	NA	8(6–12)	NA	0.82 (0.78–0.85)
LAD	All AC	0.76 (0.67–0.82)	0.42	0.81 (0.75–0.86)	0.50	13(9–20)	0.15	0.86 (0.82–0.88)
	CTAC	0.77 (0.64–0.87)	0.64	0.82 (0.71–0.89)	0.79	15(9–26)	0.41	0.87 (0.83–0.89)
	RAC	0.73 (0.64–0.81)	0.36	0.80 (0.76–0.84)	0.41	11(6–21)	0.25	0.84 (0.81–0.87)
	NAC	0.70 (0.63–0.76)	NA	0.79 (0.70–0.85)	NA	9(6–13)	NA	0.80 (0.76–0.83)
LCX	All AC	0.64 (0.52–0.75)	0.97	0.91 (0.83–0.95)	0.40	18(10–32)	0.33	0.85 (0.82–0.88)
	CTAC	0.65 (0.45–0.81)	0.34	0.89 (0.76–0.96)	0.61	15(7–33)	0.34	0.85 (0.82–0.88)
	RAC	0.64 (0.53–.075)	0.97	0.92 (0.83–0.96)	0.41	20(8–52)	0.33	0.84 (0.80–0.87)
	NAC	0.64 (0.52–0.75)	NA	0.86 (0.76–0.92)	NA	11(6–20)	NA	0.82 (0.78–0.85)
RCA	All AC	0.73 (0.63–0.80)	0.10	0.87 (0.82–0.91)	<0.001	18(11–29)	0.003	0.88 (0.85–0.91)
	CTAC	0.71 (0.54–0.84)	0.12	0.88 (0.79–0.93)	0.003	18(9–36)	0.23	0.89 (0.85–0.91)
	RAC	0.74 (0.67–0.81)	0.65	0.87 (0.82–0.90)	<0.001	19(10–37)	0.003	0.87 (0.85–0.91)
	NAC	0.82 (0.73–0.88)	NA	0.61 (0.51–0.71)	NA	7(5–10)	NA	0.79 (0.75–0.82)

*The p-values of CTAC and RAC were compared with the NAC data in the CTAC or RCA studies, separately*.

*Data in parentheses are 95% CIs*.

*AC, attenuation correction; AUC, area under receiver operating characteristic curve; CTAC, computed tomography AC; DOR, diagnostic odds ratio; LAD, left anterior descending artery; LCX, left circumflex artery; NA, not available; NAC, non-AC; PT, patient; RAC, radionuclide AC; RCA, right coronary artery*.

**Figure 2:**
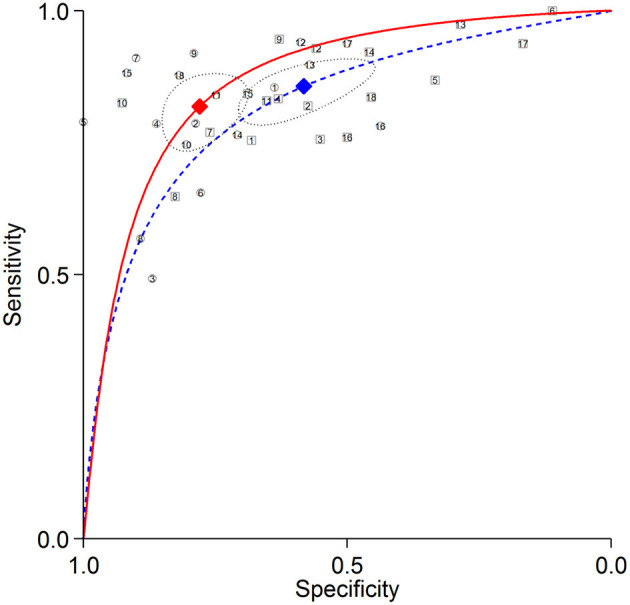
Summary ROC curves of diagnosing CAD at a patient level. Comparisons of summary ROC curves between all AC (solid line) vs. all NAC (dashed line). Each circle (AC) and square (NAC) represent an individual included study. Diamonds represent summary operating points of pooled sensitivity and specificity. AC, attenuation correction; CAD, coronary artery disease; NAC, non-AC; ROC, receiver operating characteristic.

**Figure 3:**
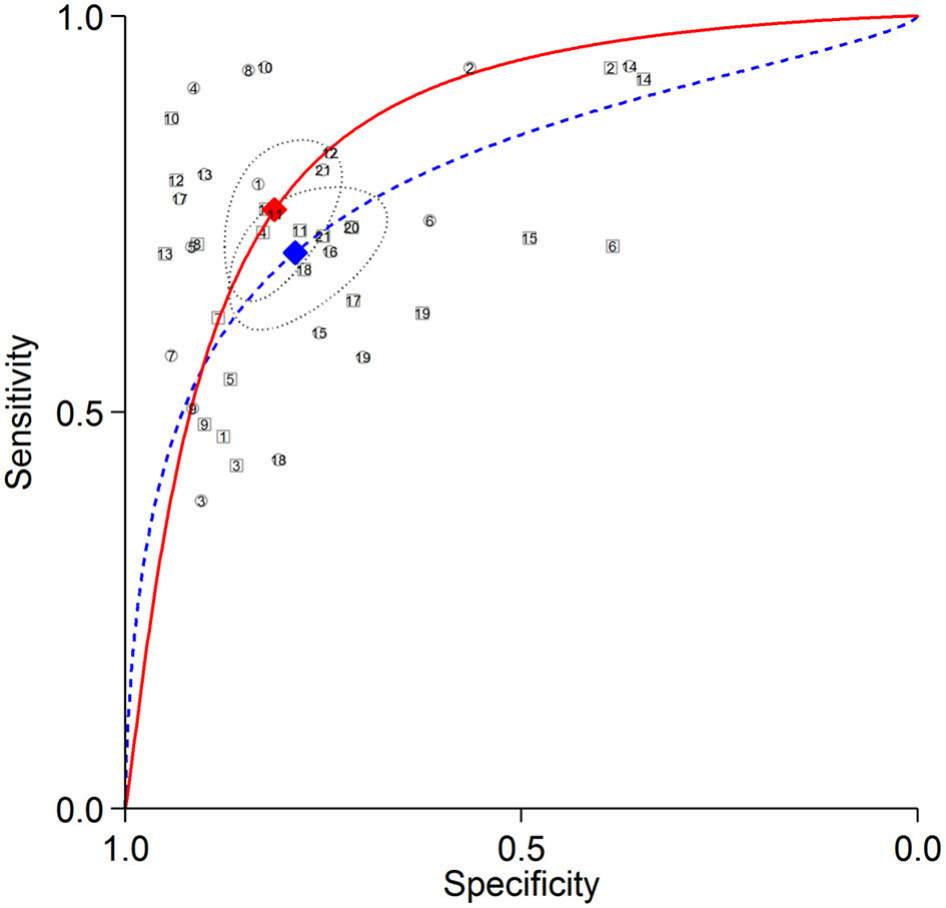
Summary ROC curves of detecting LAD stenosis. Comparisons of summary ROC curves between all AC (solid line) vs. all NAC (dashed line). Each circle (AC) and square (NAC) represent an individual included study. Diamonds represent summary operating points of pooled sensitivity and specificity. AC, attenuation correction; CAD, coronary artery disease; LAD, left anterior descending artery; NAC, non-AC; ROC, receiver operating characteristic.

**Figure 4:**
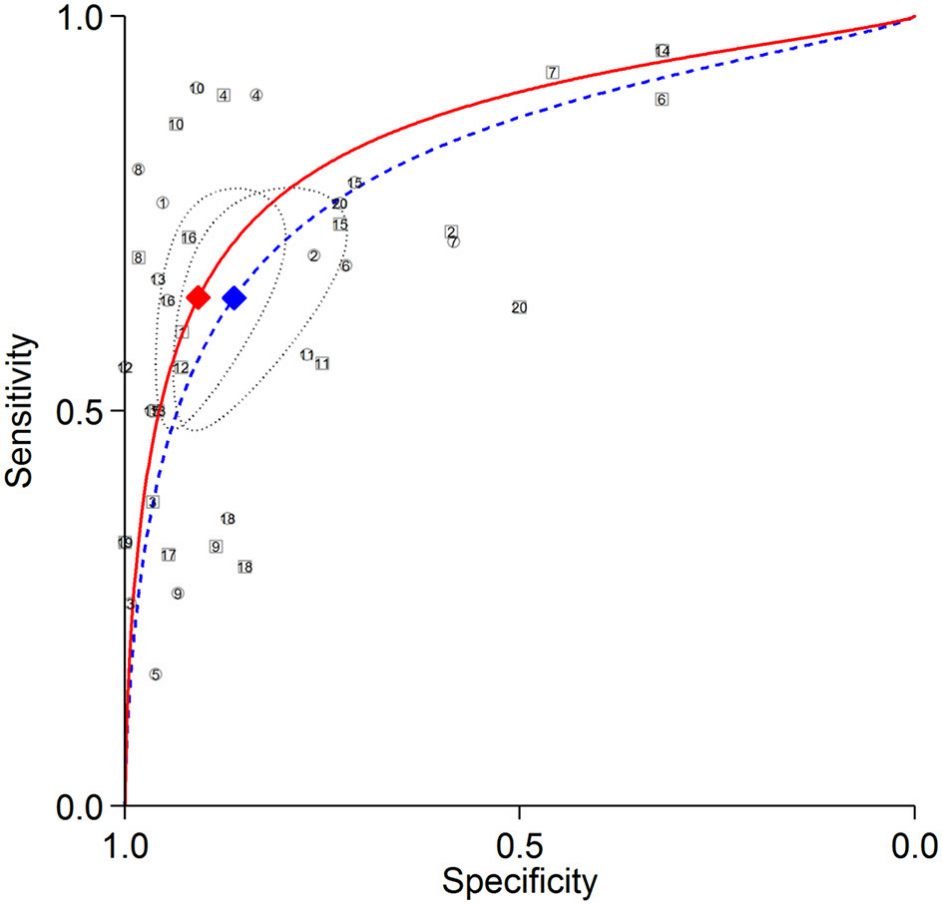
Summary ROC curves of detecting LCX stenosis. Comparisons of summary ROC curves between all AC (solid line) vs. all NAC (dashed line). Each circle (AC) and square (NAC) represent an individual included study. Diamonds represent summary operating points of pooled sensitivity and specificity. AC, attenuation correction; CAD, coronary artery disease; LCX, left circumflex artery; NAC, non-AC; ROC, receiver operating characteristic.

**Figure 5:**
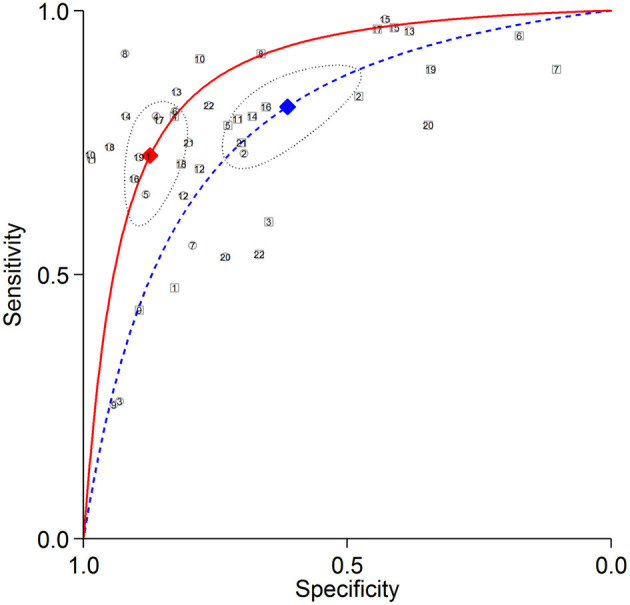
Summary ROC curves of detecting RCA stenosis. Comparisons of summary ROC curves between all AC (solid line) vs. all NAC (dashed line). Each circle (AC) and square (NAC) represent an individual included study. Diamonds represent summary operating points of pooled sensitivity and specificity. AC, attenuation correction; CAD, coronary artery disease; NAC, non-AC; RCA, right coronary artery; ROC, receiver operating characteristic.

The summary ROC curves for diagnosing CAD at a patient level and detecting LAD, LCX and RCA stenosis are shown in Figures 2–5. The AUCs for all AC, CTAC, RAC, and all NAC were 0.87 (0.84–0.89), 0.86 (0.83–0.89), 0.87 (0.84–0.90), and 0.82 (0.78–0.85), respectively, when diagnosing CAD at a patient level; 0.86 (0.82–0.88), 0.87 (0.83–0.89), 0.84 (0.81–0.87), and 0.80 (0.76–0.83) when detecting LAD stenosis; 0.85 (0.82–0.88), 0.85 (0.82–0.88), 0.84 (0.80–0.87), and 0.82 (0.78–0.85) when detecting LCX stenosis; and 0.88 (0.85–0.91), 0.89 (0.85–0.91), 0.87 (0.85–0.91), and 0.79 (0.75–0.82) when detecting RCA stenosis.

There was no significant difference in sensitivity between AC (including all AC, CTAC, and RAC) and NAC. There were significant differences in specificity between AC and NAC in diagnosing CAD at a patient level and detecting RCA stenosis, however no significant differences were noted in detecting LAD and LCX stenosis. When the diagnostic value was represented by DOR, significant differences between AC and NAC were also noted in diagnosing CAD at a patient level and detecting RCA stenosis.

We also analyzed the pooled diagnostic performance of studies with an interval between the index MPI and CAG within 3 months. 16 studies were included in the analysis and showed the same result as the whole meta-analysis with significant improvement of specificity and DOR after AC in diagnosing CAD at a patient level and detecting RCA stenosis. There were no significant differences in detecting LAD and LCX stenosis, either in sensitivity, specificity or DOR. The pooled estimates representing diagnostic performance of studies with interval between MPI and CAG within 3 months are shown in Supplementary Table 1.

### CTAC vs. RAC

The results of subgroup analysis of the type of AC were similar to those of the included studies as a whole (details are shown in Supplementary Table 2). There were no significant differences in pooled sensitivities between AC and NAC in the CTAC and RAC subgroups, or in diagnosing CAD at a patient level and detecting LAD, LCX, or RCA stenosis. Increased specificity after AC was noted in diagnosing CAD at a patient level and detecting RCA stenosis. However, a significant difference was noted only in the CTAC subgroup, but not in the RAC subgroup when diagnosing CAD at a patient level. Significant differences were noted in both CTAC and RAC subgroups when detecting RCA stenosis. Increased DOR after AC in both subgroups was noted in diagnosing CAD at a patient level and in detecting LAD, LCX, and RCA stenosis. However, a significant difference was noted only in the RAC subgroup when detecting RCA stenosis. Summary ROC curves are showed in Supplementary Figure 1. Better AUCs were noted in both the CTAC and RAC subgroups. The differences in AUCs were most obvious in detecting RCA stenosis in both the CTAC and RAC subgroups.

### ^201^Tl vs. ^99m^Tc

Subgroup analysis of the type of radiotracer was only done in detecting LAD, LCX and RCA stenosis, because only two studies used ^201^Tl as the tracer and also provided data of diagnosing CAD at a patient level (14, 30). The two studies which used dual tracers were classified into the ^99m^Tc subgroup. Ficaro et al. used a ^99m^Tc/^201^Tl stress/rest protocol, and it is well-known that the major effect of a stress MPI protocol is seen when diagnosing CAD (19). Of the 112 patients in Links' study, only 16 used ^201^Tl as the emission tracer (27). Significant improvements in specificity after AC were noted in both ^201^Tl and ^99m^Tc subgroups only when detecting RCA stenosis. DOR showed significant improvement after AC only in the ^99m^Tc subgroup (*p* = 0.012) when detecting RCA stenosis. There was an improvement in DOR in the ^201^Tl subgroup when detecting RCA stenosis, but it was not statistically significant (*p* = 0.24). Detailed data are shown in Supplementary Table 3, and summary ROC curves are shown in Supplementary Figure 2.

### Definition of CAD, 50% vs. 70% Stenosis

The results of subgroup analysis of the definition of CAD of either 50%, 70% stenosis were similar to those of the included studies as a whole (details are shown in Supplementary Table 4).

Increased specificity after AC in both subgroups was noted in diagnosing CAD at a patient level and in detecting LAD, LCX, and RCA stenosis. However, a significant difference was noted only in the 70% stenosis subgroup when diagnosing CAD at a patient level. Significant differences were noted in both 50% and 70% stenosis subgroups when detecting RCA stenosis. Increased DOR after AC in both subgroups was noted in diagnosing CAD at a patient level and in detecting LAD, and RCA stenosis. However, significant differences were noted only in 70% stenosis subgroup when diagnosing CAD at a patient level and RCA stenosis. Borderline significance (*p* = 0.05) was noted in the 50% stenosis subgroup when detecting RCA stenosis.

## Discussion

This study showed that AC significantly improved the specificity of detecting RCA disease compared to NAC. Significant improvements were also seen in the subgroup analyses of CTAC vs. NAC and RAC vs. NAC, however no significant differences in sensitivity were noted between all AC, CTAC, RAC, and NAC. Regarding the DOR in the detection of RCA stenosis, significant differences between all AC vs. NAC and RAC vs. NAC were noted. In addition, there was no significant change in the diagnostic performance after AC for LAD and LCX stenosis.

The latest meta-analysis evaluating the diagnostic performance of MPI was conducted by Xu et al. (42). They performed a meta-analysis evaluating the diagnostic performance of cardiac magnetic resonance (CMR), MPI and positron emission tomography (PET) and reported that MPI had a pooled sensitivity of 0.83 and a pooled specificity of 0.77. Xu also performed a vessel-based analysis, in which MPI showed a pooled sensitivity of 0.71 and specificity of 0.84 at a vessel level. Only 6.7% of the included articles used MPI with AC. However, no definite comparisons between the diagnostic performance of MPI with or without AC were performed, and the diagnostic accuracy in each diseased coronary artery was not mentioned. We hypothesized that the relatively lower specificity of MPI comparing with CMR and PET can be improved after AC.

Nudi et al. evaluated the diagnostic performance of MPI specifically using a cadmium-zinc-telluride (CZT) camera, and reported that CZT-MPI had satisfactory sensitivity of 0.84, but only a suboptimal specificity of 0.69 for diagnosing angiographically significant CAD (43). The suboptimal specificity may be related to attenuation artifacts. Only two of their included studies used a CZT camera equipped with CT, and the pooled estimates were similar to the NAC results of the current meta-analysis. We hypothesized that additional CTAC would also improve the specificity of CZT-MPI. With the development of newer cameras, the CT in SPECT/CT can also perform anatomic imaging of coronary arteries. Combining anatomic and functional hybrid imaging can overcome the limitations inherent to anatomic and functional testing alone, and allow for a more accurate diagnosis and guidance for interventions in patients with CAD (11, 44).

Takx et al. conducted a meta-analysis to evaluate the diagnostic accuracy of MPI, echocardiography, CMR, PET, and CT compared with invasive CAG with fractional flow reserve (FFR) for the diagnosis of hemodynamically significant CAD. Stress MPI showed a pooled sensitivity of 0.74 and pooled specificity of 0.79 at a patient level, and a pooled sensitivity of 0.61 and pooled specificity of 0.84 at a vessel level (45). In their meta-analysis, four studies used AC, five studies did not use AC, and one study did not specify whether AC was used. Comparisons between the diagnostic performance of MPI with or without AC and the diagnostic accuracy in each diseased coronary artery were still not performed. Danad et al. conducted a meta-analysis evaluating the diagnostic performance of MPI, stress echocardiography, invasive CAG, coronary computed tomography angiography (CCTA), FFR derived from CCTA, and CMR when directly compared with FFR done during CAG as the reference standard. MPI showed a pooled sensitivity of 0.70 and pooled specificity of 0.78. Danad also performed a vessel-based analysis, in which MPI showed a pooled sensitivity of 0.57 and pooled specificity of 0.75 at a vessel level (46). However, the diagnostic performance of detecting each stenosed coronary artery was still not mentioned. There was low sensitivity in vessel-based analysis compared with the current study. This may be because only six studies (282 patients) were included and with a prevalence of multi-vessel CAD ranging from 28% to 100%. Multi-vessel CAD might be missed by MPI due to balanced ischemia; however, detailed imaging protocols (such as AC or NAC) were not mentioned in data extraction. Danad et al. also performed the prospective PACIFIC trial, evaluating the diagnostic accuracy of CCTA, MPI, and PET compared with FFR. MPI showed a relatively low sensitivity of 0.57 and a very high specificity of 0.94. They claimed that the high specificity may be due to the use of CTAC (47). After applying the real gold-standard (FFR), the sensitivity of MPI seemed to be lower than using CAG as reference alone.

Only one study used CZT detector was included in our meta-analysis. CZT cameras had high spatial resolution, photon sensitivity, better image quality and can provide more accurate depiction of myocardial perfusion. Although, there were different attenuation patterns between CZT and traditional SPECT scanners (48) and the diagnostic performance of the CZT camera was already great without AC (49), the application of AC can also improve diagnostic performance, regarding specificity (39).

No previous meta-analysis has evaluated the diagnostic performance of MPI in the three coronary arteries individually. Differences in the diagnostic performance between patient level and vessel level have been shown in several studies (46, 50). Contemporary non-invasive imaging should not only focus on diagnosing CAD as a whole, but also evaluate the vascular distribution of disease (51). The diagnostic performance of MPI of the three coronary arteries can provide information on the burden of ischemia in CAD.

Both CTAC and RAC significantly improved the specificity of detecting RCA stenosis, however only CTAC significantly improved the specificity of diagnosing CAD at a patient level. Recently, with the development of new collimators, reconstruction methods and imaging protocols, the CTAC radiation dose has been significantly reduced while preserving image quality and quantitative measurements (52, 53). The dose reduction is greatest for larger patients (54), in whom AC is most commonly required (14).

In our subgroup analysis of different tracers, although a significant improvement in specificity after AC was noted in both subgroups when detecting RCA stenosis, there was only a significant improvement in DOR after AC in the ^99m^Tc subgroup. The effects of attenuation are more intense at lower energies (10), such as with ^201^Tl. Therefore, it is commonly assumed that attenuation artifacts may not be as much of an issue when using ^99m^Tc as a tracer in MPI. However, in our study, AC significantly improved specificity in both subgroups and improved DOR only in the ^99m^Tc subgroup when detecting RCA stenosis. PET was generally considered to be a better modality for detection of CAD, AC was applied inherently (55). Consequently, we recommend routine AC when performing MPI, regardless of which type of tracer or whether ECG-gated is used.

Our previous meta-analysis focused on different imaging techniques (AC vs. NAC) in MPI, but only in diagnosing the existence of CAD at a patient level (56). The accuracy of identifying territories in jeopardy is clinically important, however it has not been reported before. This is the first study to report the sensitivity, specificity, DOR and AUC of MPI with and without AC in different coronary arteries. We included 22 studies with 2,608 patients which evaluated both AC and NAC results in the same participants. Statistical comparisons between two different index tests require direct comparisons between the same individual data. No publication bias was identified among the selected studies, and therefore the results can be considered robust.

This study has several limitations. First, heterogeneity was noted among the included studies. In order to generalize our results, we have to broad the included studies to all studies that evaluate the effect of AC in individual coronary artery stenosis, there might be some substantial heterogeneities between the included studies. However, all of the included studies followed the guidelines of the American Society of Nuclear Cardiology and the American Heart Association, and the diagnostic performance of MPI remained acceptable and stable regardless of the protocols. Second, the included studies mainly used visual analysis and lacked a standard semiquantitative method. Third, verification bias existed because the results of MPI, which may have affected referral to invasive CAG. Only one study performed CAG irrespective of the MPI results (27). Fourth, non-perfusion parameters or ECG-gated information was not routinely used for interpretation, which may have underestimated the diagnostic accuracy of the study. Therefore, the true diagnostic performance of MPI could be better than the reported estimates after combining these parameters. Also, the information from ECG-gated and AC cannot be replaced by each other (40, 57). Fifth, none of the included studies used FFR analysis in addition to quantitative CAG as the reference standard. However, quantitative CAG alone does not predict the functional significance of coronary lesions or micro-vascular disease (58). Sixth, subgroup analysis to define which category of patients, such as gender or body habitus, may benefit most from AC could not be established, due to lack of individual data from the included articles. Seventh, only one CZT study was included and there is a concern to generalize our findings in this newer scanner (39). However, Caobelli's results were concordant to our main findings. Finally, differences between the AUCs of AC and NAC could not be calculated because of a lack of information regarding the relationships between AC and NAC in patients with and without CAD. Therefore, we calculated DORs as acceptable substitutes.

## Conclusion

The results of this study suggest that AC should be applied to MPI to improve the diagnosis of CAD, and that AC MPI can improve the specificity of detecting RCA stenosis.

## Data Availability Statement

The original contributions presented in the study are included in the article/Supplementary Material, further inquiries can be directed to the corresponding author/s.

## Author Contributions

J-YH, K-LC, and Y-WW conceived and designed this study. J-YH, C-KH, and R-FY were responsible for analysis and interpretation of the data. J-YH and C-KH were responsible for writing the manuscript. K-LC and Y-WW took part in manuscript review and revision. All authors contributed to the article and approved the submitted version.

## Funding

This study was partly supported by grants MOST 107-2314-B-418-006-MY3, MOST 108-2314-B-418-002-MY3 (from Y-WW), MOST 106-2314-B-002−049-MY3, MOST 109-2314-B-002-253, and MOST 110-2314-B-002-141 (from J-YH) from the Ministry of Science and Technology of Taiwan. The funders had no role in study design, data collection and analysis, decision to publish, or preparation of the manuscript.

## Conflict of Interest

The authors declare that the research was conducted in the absence of any commercial or financial relationships that could be construed as a potential conflict of interest.

## Publisher's Note

All claims expressed in this article are solely those of the authors and do not necessarily represent those of their affiliated organizations, or those of the publisher, the editors and the reviewers. Any product that may be evaluated in this article, or claim that may be made by its manufacturer, is not guaranteed or endorsed by the publisher.
