# Supplementary material for: Diagnostic Effect of Attenuation Correction in Myocardial Perfusion Imaging in Different Coronary Arteries: A Systematic Review and Meta-Analysis
Source: Front Cardiovasc Med. 2021 Oct 12;8:756060. doi: 10.3389/fcvm.2021.756060 (PMC8545877; doi:10.3389/fcvm.2021.756060)
Supplement: Supplementary file 1 [file Image_1.PDF]

Supplementary Figure 1

Summary ROC curves of the CTAC subgroup (red) and RAC subgroup (blue). Comparisons of summary ROC curves between AC (solid line) versus NAC (dashed line) in the CTAC and RAC subgroups.

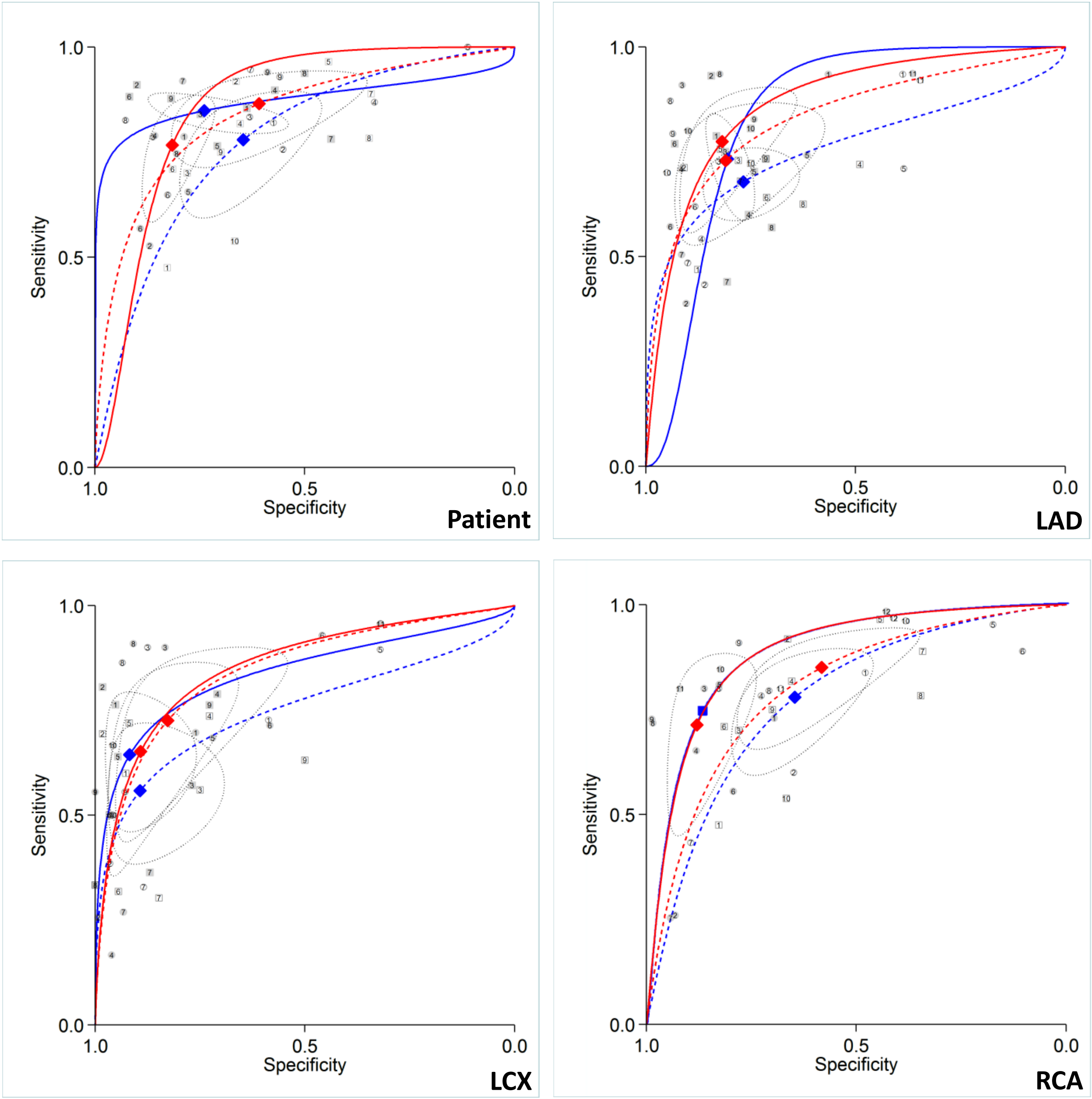

CTAC: computed tomography attenuation correction; LAD: left anterior descending artery; LCX: left circumflex artery; RAC: radionuclide attenuation correction; RCA: right coronary artery; ROC: receiver operating characteristic
