# Supplementary material for: Diagnostic Effect of Attenuation Correction in Myocardial Perfusion Imaging in Different Coronary Arteries: A Systematic Review and Meta-Analysis
Source: Front Cardiovasc Med. 2021 Oct 12;8:756060. doi: 10.3389/fcvm.2021.756060 (PMC8545877; doi:10.3389/fcvm.2021.756060)
Supplement: Supplementary file 2 [file Image_2.PDF]

Supplementary Figure 2

Summary ROC curves of the thallium-201 subgroup (red) and technetium-99m subgroup (blue). Comparisons of summary ROC curves between AC (solid line) versus NAC (dashed line) in the thallium-201 and technetium-99m subgroups.

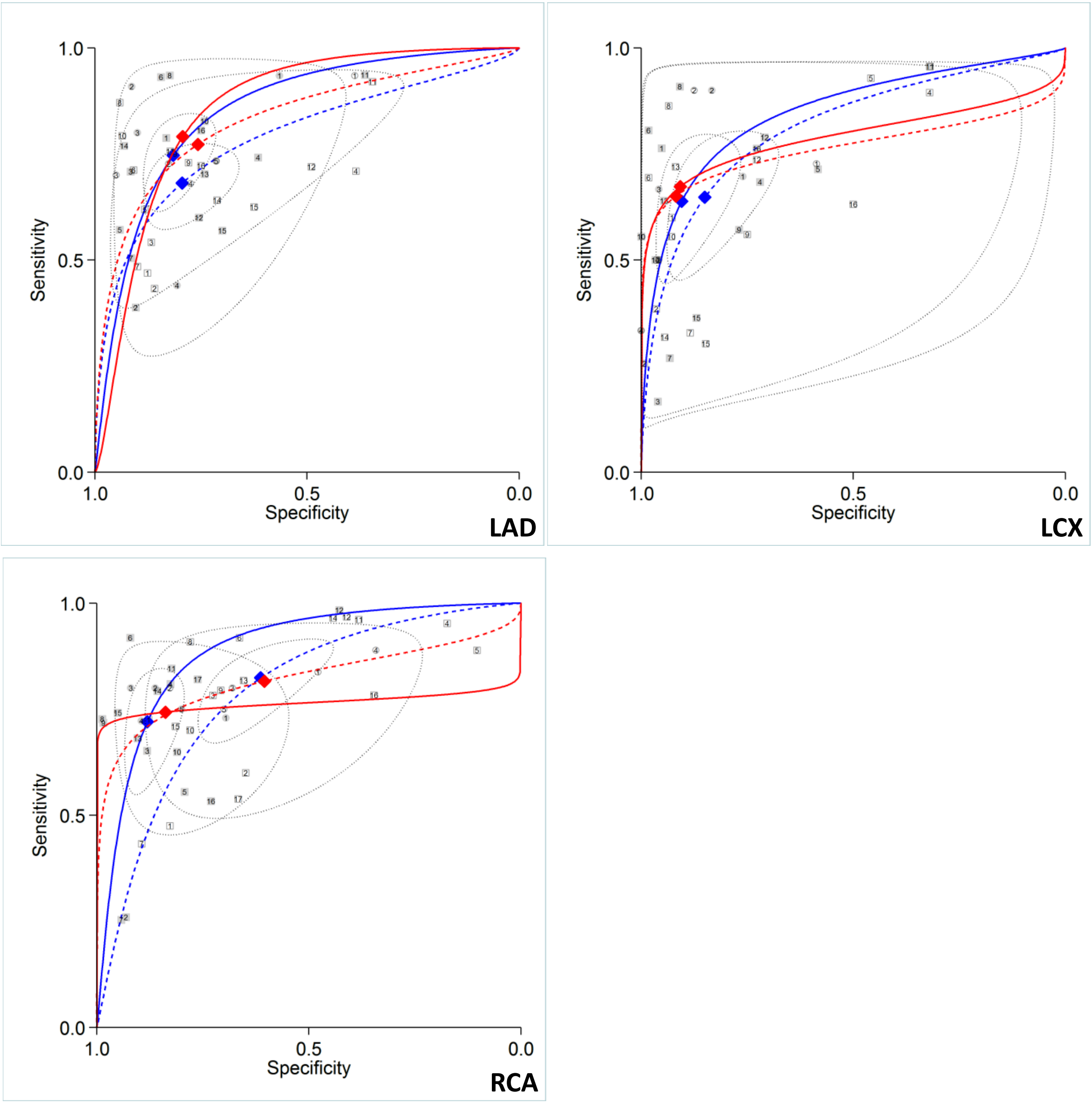

AC: attenuation correction; LAD: left anterior descending artery; LCX: left circumflex artery; NAC: non-AC; RCA: right coronary artery; ROC: receiver operating characteristic
