# Supplementary material for: Diagnostic Effect of Attenuation Correction in Myocardial Perfusion Imaging in Different Coronary Arteries: A Systematic Review and Meta-Analysis
Source: Front Cardiovasc Med. 2021 Oct 12;8:756060. doi: 10.3389/fcvm.2021.756060 (PMC8545877; doi:10.3389/fcvm.2021.756060)
Supplement: Supplementary file 3 [file Table_1.PDF]

Supplementary Table 1. Diagnostic Performance of MPI comparing with CAG within 3 months, Pooled Sensitivity, Specificity, Diagnostic OR and Area Under the Receiver Operating Characteristic Curve of All AC, and NAC in Diagnosing CAD at a Patient Level and Detecting LAD, LCX and RCA Stenosis.

		**Sensitivity**	**P value**	**Specificity**	**P value**	**DOR**	**P value**	**AUC**
**PT**	All AC	0.83 (0.77-0.87)	0.74	0.76* (0.69-0.82)	0.026	15* (10-23)	0.035	0.86 (0.83-0.89)
	All NAC	0.84 (0.78-0.88)	NA	0.62 (0.52-0.72)	NA	8 (6-12)	NA	0.82 (0.79-0.85)
**LAD**	All AC	0.76 (0.67-0.83)	0.44	0.80 (0.75-0.85)	0.98	13 (8-21)	0.50	0.85 (0.82-0.88)
	NAC	0.70 (0.63, 0.77)	NA	0.81 (0.73, 0.88)	NA	10 (6, 17)	NA	0.81 (0.78 - 0.85)
**LCX**	All AC	0.62 (0.50-0.73)	0.67	0.92 (0.86-0.96)	0.49	19 (9-41)	0.40	0.87 (0.84-0.90)
	NAC	0.59 (0.49-0.68)	NA	0.89 (0.82-0.94)	NA	12 (6-25)	NA	0.79 (0.75-0.82)
**RCA**	All AC	0.71 (0.62-0.79)	0.20	0.88* (0.83-0.92)	<0.001	19* (11-33)	0.008	0.88 (0.85-0.91)
	NAC	0.80 (0.70-0.87)	NA	0.65 (0.56-0.74)	NA	7 (5-11)	NA	0.79 (0.75-0.82)

* Data in parentheses are 95% CIs.

* AC: attenuation correction; AUC: area under *receiver operating characteristic curve;* CTAC: computed tomography AC: DOR: diagnostic odds ratio; LAD: left anterior descending artery; LCX: left circumflex artery; NA: not available; NAC: non-AC; PT: patient; RAC: radionuclide AC; RCA: right coronary artery

*P<0.05
