# Supplementary material for: Diagnostic Effect of Attenuation Correction in Myocardial Perfusion Imaging in Different Coronary Arteries: A Systematic Review and Meta-Analysis
Source: Front Cardiovasc Med. 2021 Oct 12;8:756060. doi: 10.3389/fcvm.2021.756060 (PMC8545877; doi:10.3389/fcvm.2021.756060)
Supplement: Supplementary file 4 [file Table_2.PDF]

Supplementary Table 2. Subgroup analysis of CTAC vs. RAC.

Diagnostic performance of MPI, pooled sensitivity, specificity, diagnostic OR and area under the receiver operating characteristic curve of AC and NAC in diagnosing CAD at a patient level and detecting LAD, LCX and RCA stenosis in CTAC and RAC subgroups.

			**Sensitivity**	**Specificity**	**DOR**	**AUC**
All patients	CTAC	AC	0.77 (0.66-0.85)	0.82* (0.75-0.86)	15 (10-22)	0.86 (0.83-0.89)
		NAC	0.87 (0.78-0.92)	0.61 (0.44-0.75)	10 (5-18)	0.84 (0.80-0.87)
	RAC	AC	0.85 (0.82-0.88)	0.74 (0.62-0.83)	16 (8-33)	0.87 (0.84-0.90)
		NAC	0.86 (0.77-0.84)	0.57 (0.45-0.68)	8 (6-11)	0.81 (0.77-0.84)
LAD vessel	CTAC	AC	0.77 (0.64-0.87)	0.82 (0.71-0.89)	15 (9-26)	0.87 (0.83-0.89)
		NAC	0.73 (0.60-0.83)	0.81 (0.66-0.90)	11 (6-23)	0.83 (0.80-0.86)
	RAC	AC	0.73 (0.64-0.81)	0.80 (0.76-0.84)	11 (6-21)	0.84 (0.81-0.87)
		NAC	0.68 (0.62-0.73)	0.77 (0.68-0.83)	7 (4-11)	0.76 (0.72-0.80)
LCX vessel	CTAC	AC	0.65 (0.45-0.81)	0.89 (0.76-0.96)	15 (7-33)	0.85 (0.82-0.88)
		NAC	0.73 (0.54-0.86)	0.83 (0.65-0.92)	13 (6-26)	0.84 (0.81-0.87)
	RAC	AC	0.64 (0.53-.075)	0.92 (0.83-0.96)	20 (8-52)	0.84 (0.80-0.87)
		NAC	0.56 (0.45-0.67)	0.89 (0.78-0.95)	11 (4-26)	0.75 (0.71-0.79)
RCA vessel	CTAC	AC	0.71 (0.54-0.84)	0.88* (0.79-0.93)	18 (9-36)	0.89 (0.85-0.91)
		NAC	0.85 (0.73-0.92)	0.58 (0.42-0.73)	8 (5-13)	0.80 (0.76-0.83)
	RAC	AC	0.74 (0.67-0.81)	0.87* (0.82-0.90)	19* (10-37)	0.87 (0.85-0.91)
		NAC	0.78 (0.66-0.86)	0.65 (0.52-0.75)	6 (4-11)	0.78 (0.74-0.81)

* AC: attenuation correction; AUC: area under receiver operating characteristic curve; CTAC: computed tomography AC: DOR: diagnostic odds ratio; LAD: left anterior descending artery; LCX: left circumflex artery; NAC: non-AC; PT: patient; RAC: radionuclide AC; RCA: right coronary artery

*: $p<0.05$
