# Supplementary material for: Diagnostic Effect of Attenuation Correction in Myocardial Perfusion Imaging in Different Coronary Arteries: A Systematic Review and Meta-Analysis
Source: Front Cardiovasc Med. 2021 Oct 12;8:756060. doi: 10.3389/fcvm.2021.756060 (PMC8545877; doi:10.3389/fcvm.2021.756060)
Supplement: Supplementary file 5 [file Table_3.PDF]

Supplementary Table 3. Subgroup analysis of ^{99m}Tc vs. ^{201}Tl.

Diagnostic performance of MPI, pooled sensitivity, specificity, diagnostic OR and area under the receiver operating characteristic curve of All AC and NAC in diagnosing CAD at a patient level and detecting LAD, LCX and RCA stenosis in ^{99m}Tc and ^{201}Tl subgroups.

			**Sensitivity**	**Specificity**	**DOR**	**AUC**
All patients	^{99m}Tc	AC	0.82 (0.75-0.86)	0.79* (0.72-0.84)	16* (11-25)	0.84 (0.84-0.90)
		NAC	0.85 (0.80-0.90)	0.60 (0.50-0.69)	9 (6-12)	0.82 (0.79-0.85)
LAD vessel	^{201}Tl	AC	0.79 (0.57-0.91)	0.79 (0.64-0.89)	14 (5-43)	0.86 (0.82-0.89)
		NAC	0.77 (0.61-0.88)	0.76 (0.55-0.89)	11 (5-22)	0.83 (0.80-0.86)
	^{99m}Tc	AC	0.75 (0.65-0.82)	0.82 (0.75-0.87)	13 (8-20)	0.85 (0.82-0.88)
		NAC	0.68 (0.60-0.75)	0.79 (0.70-0.86)	8 (5-13)	0.79 (0.75-0.82)
LCX vessel	^{201}Tl	AC	0.67 (0.47-0.83)	0.91 (0.70-0.98)	20 (5-81)	0.79 (0.75-0.82)
		NAC	0.65 (0.44-0.82)	0.92 (0.62-0.99)	21 (4-121)	0.77 (0.73-0.80)
	^{99m}Tc	AC	0.64 (0.50-0.76)	0.90 (0.82-0.95)	17 (9-33)	0.85 (0.81-0.88)
		NAC	0.65 (0.51-0.77)	0.85 (0.73-0.92)	10 (6-19)	0.81 (0.78-0.85)
RCA vessel	^{201}Tl	AC	0.74 (0.62-0.84)	0.84* (0.73-0.91)	15 (6-36)	0.76 (0.72-0.80)
		NAC	0.82 (0.78-0.85)	0.60 (0.42-0.76)	7 (3-16)	0.82 (0.78-0.85)
	^{99m}Tc	AC	0.72 (0.60-0.82)	0.88* (0.82-0.92)	19* (11-33)	0.89 (0.86-0.91)
		NAC	0.82 (0.72-0.90)	0.61 (0.49-0.72)	7 (5-11)	0.79 (0.75-0.82)

* AC: attenuation correction; AUC: area under receiver operating characteristic curve; CTAC: computed tomography AC: DOR: diagnostic odds ratio; LAD: left anterior descending artery; LCX: left circumflex artery; NAC: non-AC; PT: patient; RAC: radionuclide AC; RCA: right coronary artery

*: $p<0.05$
