# Supplementary material for: Diagnostic Effect of Attenuation Correction in Myocardial Perfusion Imaging in Different Coronary Arteries: A Systematic Review and Meta-Analysis
Source: Front Cardiovasc Med. 2021 Oct 12;8:756060. doi: 10.3389/fcvm.2021.756060 (PMC8545877; doi:10.3389/fcvm.2021.756060)
Supplement: Supplementary file 6 [file Table_4.PDF]

Supplementary Table 4. Subgroup analysis of 50% vs. 70% stenosis.

Diagnostic performance of MPI, pooled sensitivity, specificity, diagnostic OR and area under the receiver operating characteristic curve of All AC, and NAC in diagnosing CAD at a patient level and detecting LAD, LCX and RCA stenosis in CAD definition of 50% and 70% stenosis subgroups.

			**Sensitivity**	**Specificity**	**DOR**	**AUC**
All patients	50%	AC	0.82 [0.70, 0.90]	0.80 [0.67, 0.88]	18 [8, 40]	0.88 [0.85 - 0.90]
		NAC	0.79 [0.73, 0.84]	0.65 [0.57, 0.73]	7 [5, 10]	0.79 [0.75 - 0.82]
	70%	AC	0.82* [0.77, 0.85]	0.77* [0.71, 0.82]	15* [11, 20]	0.86 [0.83 - 0.89]
		NAC	0.89 [0.83, 0.94]	0.50 [0.34, 0.67]	9 [5, 16]	0.86 [0.83 - 0.89]
LAD vessel	50%	AC	0.76 [0.63, 0.86]	0.82 [0.72, 0.89]	14 [8, 27]	0.86 [0.83 - 0.89]
		NAC	0.65 [0.54, 0.75]	0.81 [0.70, 0.89]	8 [5, 14]	0.79 [0.75 - 0.83]
	70%	AC	0.74 [0.63, 0.82]	0.79 [0.72, .85]	11 [7, 18]	0.84 [0.80 - 0.87]
		NAC	0.75 [0.67, 0.81]	0.75 [0.61, 0.85]	9 [5, 16]	0.80 [0.76 - 0.83]
LCX vessel	50%	AC	0.61 [0.42, 0.78]	0.94 [0.84, 0.98]	26 [10, 67]	0.88 [0.84 - 0.90]
		NAC	0.54 [0.38, 0.70]	0.89 [0.77, 0.95]	10 [5, 22]	0.79 [0.75 - 0.83]
	70%	AC	0.65 [0.53, 0.76]	0.86 [0.74, 0.92]	11 [6, 23]	0.80 [0.77 - 0.84]
		NAC	0.72 [0.59, 0.83]	0.81 [0.61, 0.92]	12 [5, 28]	0.81 [0.78 - 0.85]
RCA vessel	50%	AC	0.74 [0.53, 0.88]	0.87* [0.77, 0.92]	19# [9, 41]	0.89 [0.86 - 0.91]
		NAC	0.76 [0.60, 0.88]	0.66 [0.53, 0.77]	6 [4, 11]	0.77 [0.73 - 0.80]
	70%	AC	0.69* [0.64, 0.74]	0.88* [0.82, 0.93]	17* [9, 32]	0.74 [0.70 - 0.78]
		NAC	0.85 [0.77, 0.91]	0.54 [0.39, 0.68]	7 [4, 11]	0.81 [0.78 - 0.84]

AC: attenuation correction; AUC: area under receiver operating characteristic curve; CTAC: computed tomography AC: DOR: diagnostic odds ratio; LAD: left anterior descending artery; LCX: left circumflex artery; NAC: non-AC; PT: patient; RAC: radionuclide AC; RCA: right coronary artery

*: p<0.05

# p=0.05

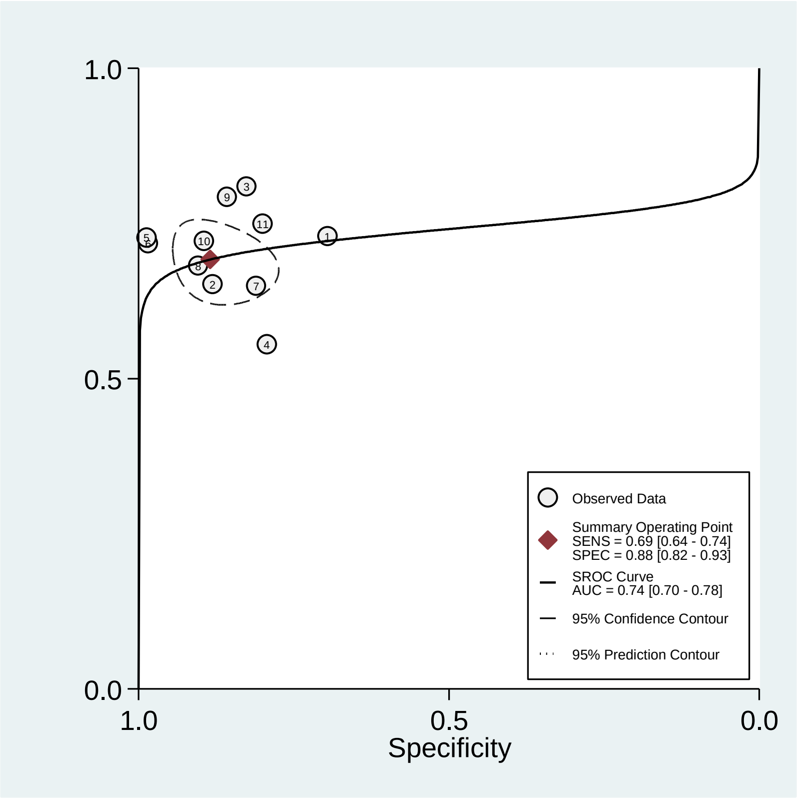
1.0
0.5
0.0
1.0
0.5
0.0
Specificity
Observed Data
Summary Operating Point
SENS = 0.69 [0.64 - 0.74]
SPEC = 0.88 [0.82 - 0.93]
SROC Curve
AUC = 0.74 [0.70 - 0.78]
95% Confidence Contour
95% Prediction Contour

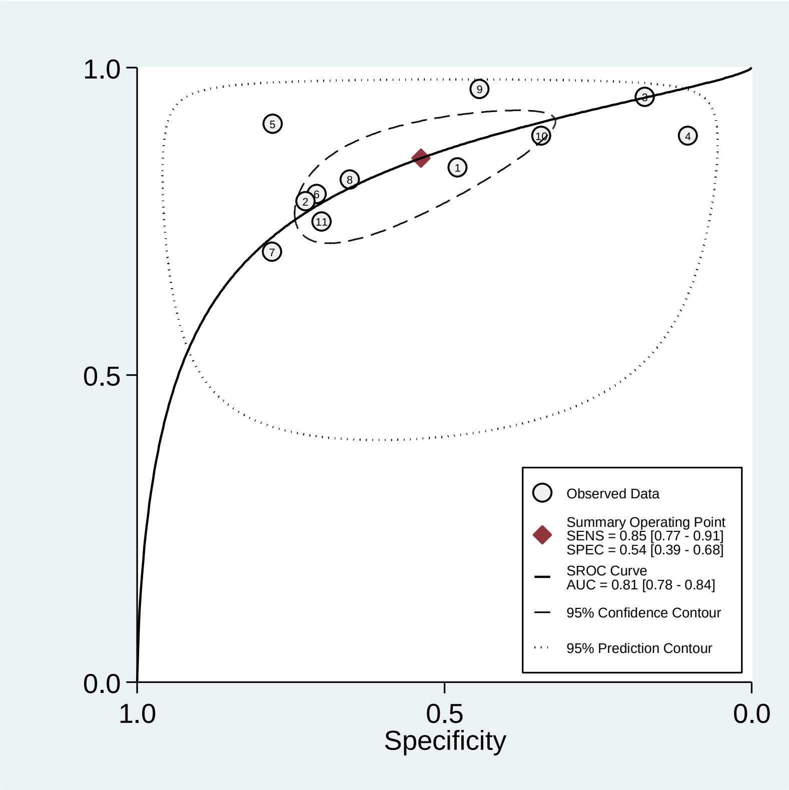
1.0
0.5
0.0
1.0
0.5
0.0
Specificity
Observed Data
Summary Operating Point
SENS = 0.85 [0.77 - 0.91]
SPEC = 0.54 [0.39 - 0.68]
SROC Curve
AUC = 0.81 [0.78 - 0.84]
95% Confidence Contour
95% Prediction Contour
